# Neglect of the complex: why psychotherapy for post-traumatic clinical presentations is often ineffective^†^

**DOI:** 10.1192/pb.bp.114.046995

**Published:** 2015-04

**Authors:** Frank M. Corrigan, Alastair M. Hull

**Affiliations:** 1 Argyll & Bute Hospital, Lochgilphead; 2 Perth Royal Infirmary, Perth

## Abstract

Evidence of efficacy in studies of post-traumatic conditions is largely derived from studies in which variables are kept to a minimum. Extrapolation of treatments from uncomplicated disorders to complex conditions may therefore be called evidence-based without being evidenced. Complex conditions with polysymptomatic presentations and extensive comorbidity are being denied proper evaluation, and patients most severely traumatised from the early stages of their development are not provided with rigorously evaluated psychotherapies because they are more difficult to study in the manner approved by research protocols. Such evidence as there is suggests that the simple extension of treatments for uncomplicated disorders is seriously inadequate. This has significant implications for health services responsible for the provision of the most efficacious treatments to those whose disorders arise from severe trauma, often very early in their life.

## Evidence-based and evidenced are not necessarily the same

Psychotherapy for post-traumatic clinical presentations is often restricted by the lack of evidence in support of approaches other than those validated for non-complex post-traumatic stress disorder (PTSD), such as cognitive–behavioural therapy (CBT)^1^ and eye movement desensitisation and reprocessing (EMDR).^2,3^ Complex PTSD has different definitions but is essentially a multifaceted presentation arising from extreme stress, usually at an early developmental level. This leads to difficulty in regulating affective arousal; alterations in attention and consciousness such as amnesia and dissociation; somatisation; chronic characterological changes; and alterations in systems of meaning.^4^ The variability in the syndromes that result means that inexact use of terminology bedevils this clinical and research area. While PTSD is a theoretical umbrella term,^5^ we use ‘complex PTSD’ in this paper to refer to complex reactions to multiple traumatic stressor exposures and experiences, usually against a background of severe disturbances in primary caregiving relationships.

Complex presentations are often excluded from studies because they do not fit neatly into the simple nosological categorisations required for research power. This means that the most severe disorders are not studied adequately and patients most affected by early trauma are often not recognised by services. Both historically and currently, at the individual as well as the societal level, dissociation from the acknowledgement of the severe impact of childhood abuse on the developing brain leads to inadequate provision of services. Assimilation into treatment models of the emerging affective neuroscience of adverse experience could help to redress the balance by shifting the focus from top-down regulation to bottom-up, body-based processing.

At present there is little regard for the subcortical generators of distress and an overemphasis on the cognitive strategies needed to manage the resulting emotions. At the institutional level this translates to a preoccupation with therapist supervision; attainment of symptom-reduction goals; invalidation of the importance of affective experience; and intolerance of clinical complexity. There are then imposed limitations on psychotherapy sessions and inadequate time and emphasis on therapeutic engagement. The concept of evidence needs to be expanded to include neuroscientific plausibility. The collection of outcome data needs to include biological data such as changes in functional imaging responses to trauma-related and apparently innocuous interpersonal stimuli. Neuroscientific plausibility can be a source of indirect evidence and affective neuroscience can be included in the rationale for novel treatments in complex PTSD. We explore this elsewhere (details available from the authors on request). However, acknowledgement of the magnitude of the problem would have severe financial implications for mental health services.

## Limitations of the evidence base for the treatment of complex PTSD

Evidence-based therapy for mental disorders is often considered to be CBT as it has been shown to be of value in reducing symptoms in many disorders. CBT has been able to accumulate evidence in part through the readiness of funding bodies to provide for research when there is likely to be some observable and measurable benefit, however clinically relevant – in terms of symptoms or functioning. If research funding is not readily accessible for complex and prolonged interventions that are clinically applied in the phase-based treatment of complex PTSD, it is easy to arrive at the false conclusion that a lack of evidence for a particular therapy indicates that it is not effective. There is not, to our knowledge, any register of projects which have been refused financial support or discouraged from making a full application on the basis of cost. One example is sensorimotor psychotherapy,^6^ which has as yet little supporting evidence but is endorsed by leading international experts and is neuroscientifically credible. The costs of carrying out an outcome study of sensorimotor psychotherapy with current methodological constraints would be prohibitive. Somatic experiencing^7^ preceded sensorimotor psychotherapy as a body-based therapy for the resolution of traumatic experience and is widely used throughout the world. It also lacks the evidence base deemed necessary in those services for which rapid symptom reduction is the economically derived priority.

Both the National Institute for Health and Care Excellence (NICE) guidelines^8^ and a Cochrane review of psychological therapies for chronic PTSD in adults^9^ concluded that EMDR and trauma-focused CBT are effective in clinician-rated symptom reduction, although there was evidence of greater dropout in active treatment groups. The authors of both also considered the evidence available to them to be of low quality. A Cochrane review of psychological therapies for people with borderline personality disorder^10^ concluded that dialectical behaviour therapy (DBT) was effective in reducing anger and parasuicidality and in improving general mental health, but it did not appear to be more likely than treatment as usual to keep people in therapy. The authors considered that none of the treatments they studied for borderline personality disorder had ‘a very robust evidence base’.

## Consequences of accepting ‘evidence-based’ as ‘evidenced’

If CBT and/or DBT were effective for 100% of patients with complex trauma sequelae there would be no need for additional therapeutic approaches. To illustrate our contention that this may not be the case, a relevant paper^11^ recommended to us as methodologically sound has been selected. This helps to clarify the answer to the 100% resolution question in regard to CBT. In this paper by McDonagh and colleagues,^11^ exclusion criteria were: use of medication with significant autonomic nervous system effects; dissociative identity disorder; current alcohol or drug misuse; presence of active suicidality or a history of two or more suicide gestures or attempts in the preceding year. Women were also excluded from the study if they were in a relationship with an abusive partner, a situation unfortunately all too common in this clinical population. Although the eventual study group had experienced multiple traumas, those who completed treatment were middle-aged, well educated and in employment. Many of the patients encountered in general psychiatric practice do not fit this profile. Many of those who present clinically with a history of complex PTSD have been attempting to manage their distress through one – or more likely a combination – coping strategies, for instance self-harm, alcohol/drug misuse, eating disorders, or other behaviours designed to limit their sudden shifts out of the ‘window of tolerance’.^12^ The efforts to achieve physiological regulation themselves then lead to further difficulties. Because treatment studies in general dislike comorbidity, the evidence on treatment approaches to multiple, coexisting and complex problems is limited.

As well as the exclusion of people who need therapy – such as those who are chronically suicidal as a result of early trauma – there was evidence of a problem with dropouts from the study. This was most evident with CBT (41%) and required the discharge of the random assignment process to get sufficient numbers into the CBT group. The post-treatment analysis applying intention-to-treat showed no significant difference in the numbers no longer meeting PTSD criteria: 28% for CBT (*n* = 8); 32% for present-centred therapy (*n* = 7); 17% for the waiting list (*n* = 4). So of the 200+ patients who met the criteria for complex PTSD following childhood sexual abuse, 74 were included in the study and 8 got better with CBT compared with 4 on the waiting list. This falls well short of a 100% recovery criterion which would support the restriction of training to CBT, and raises serious questions about CBT being the core treatment modality provided for complex post-traumatic presentations.

For the completers only (i.e. ignoring those who dropped out) both treatment groups improved significantly compared with the waiting list and both showed sustained improvements at 6 months. CBT therefore had clear and demonstrable benefits for some female childhood sexual abuse survivors. However, patients were more likely to stay in present-centred therapy, in which the therapists were required to be genuine, empathic and non-judgemental.

This is only one methodologically sound study of a selected population but it is of interest that the problem with the dropout rate has been previously observed for clinical practice in the ‘real world’,^13^ in which many psychologists trained in CBT were found to be reluctant to use imaginal exposure.^14^ There is a striking discrepancy between recommended best evidence-based practice for PTSD and actual clinical practice.^15^ The underlying reasons for this discrepancy are likely to be complex but may reflect the clinician’s view of the tolerability of the therapy for both patient and practitioner. Prolonged exposure may be necessary for some who prefer to spend the hours on slow adaptation rather than to go with the rapid information processing available in non-exposure treatment protocols such as EMDR.^2^ However, EMDR cannot be applied in complex PTSD with strict adherence to the standard protocol used in non-complex PTSD without a high risk of increasing dysregulation. For the multiple traumatic events and experiences of the kind commonly encountered by victims of child sexual abuse, prolonged exposure is unlikely to work in the lifetime of the patient.

The context-dependent unhitching of stimulus and response can occur without any impact on the stored representation of the unconditioned stimulus.^16^ If the unconditioned stimulus involves a body memory from being raped at 3 years old, it may be possible to reduce the distress related to adult sexual activity without having any impact on the stored and readily triggered pain, rage, terror, shame, abandonment, isolation, worthlessness, hopelessness, helplessness or survival terror. Also unaffected will be the dissociative defences which helped the child to survive and continue with life, apparently unscathed. The therapeutic gains are therefore helpful, but limited.

Therapists engaged in the provision of prolonged exposure may be troubled by ‘feelings of helplessness’.^17^ So if the therapists feel helpless, they then need to spend more time in supervision, being exposed to their helplessness with a supervisor who presumably feels less helpless because he or she is supervising rather than treating. Subsequently, within systems there is then less time available to treat those patients who are willing and able to participate in the exposure therapy that even those supplying it dislike and prefer to avoid. It may also be the personal preference of clinical researchers to focus on the cognitive, as in restructuring, rather than be exposed to the realms of horror and terror, intense isolation and abandonment, excruciating pain and despair of the complex trauma survivor. If the therapist has unresolved residues of traumatic experience himself, the ability to convey the psychotherapy may be even more challenging; it is then much easier to focus on reappraisal and the reassurance that all present have survived and prospered.

## Dropouts from DBT

Dialectical behaviour therapy provides techniques for safety and stabilisation of borderline personality disorders^18^ and some of its elements have been adapted for dissociative disorders.^19^ In DBT emotions are recognised as an important part of human experience and there is considerable emphasis on their regulation to reduce distress. So it is interesting to see that dropout rates from DBT in the UK can increase, from an already high 52% to 88% in those with more complex presentations.^20^ Of course, not all patients with borderline personality disorder have a history of trauma or unresolved attachment and genetic and other factors may be present in some.^21^ However, between 40 and 70% of those with borderline personality disorder would also meet criteria for one of the major dissociative disorders in which trauma histories and disorganised attachment are major aetiological factors.^21^ It is surprising, but perhaps a reflection of what is considered treatable, that attachment trauma is often ignored, despite research specifying feelings of emptiness and problems in coping with abandonment as key features of borderline personality disorder.^10^ Treatment continuity may be interfered with by the behaviourist management of dissociation as a problem behaviour, which can be approached through desensitisation of present cues to past traumatic experiences.^21^ The structural dissociation model of van der Hart *et al*^22^ sees self-states that interfere with therapy as nevertheless based in the defence from the overwhelming effects of trauma. Therapists working with an ego state model in which the cooperation of aggressive protector parts is a prerequisite for continuing treatment (e.g. Paulsen^23^) have identified and delineated strategies for achieving this. It would be interesting to know whether the disregard for the original survival functions of peritraumatic and structural dissociation contributes to the high dropout from DBT. A very testable hypothesis is that people who drop out from DBT are primarily those with significant but unrecognised dissociative disorders.

## Prevalence of dissociative disorders

There is evidence that some of the complex post-traumatic disorders – including dissociative disorders – can have an impact on functioning equivalent at least to major psychotic disorders, and should be considered to be ‘serious mental illness’.^24^ Studies of the general population find a prevalence rate for dissociative identity disorder at 1–3%, whereas in psychiatric patient populations the figure is 1–5%.^25^ Those individuals are often not diagnosed as having dissociative identity disorder but receive treatment according to the most prominent signs and symptoms, and their response to treatment for depression, anxiety, panic disorder, eating disorder, substance misuse or somatoform disorders will inevitably be incomplete. Moreover, unreported or unrecognised trauma is common in psychiatric patients (details available from the authors on request). Unfortunately, in controlled trials in groups of patients presenting with these symptoms and syndromes the diagnosis of those who drop out is not reassessed. Ethical constraints would prevent attempts to acquire this information after a patient has dropped out, so there is a need to assess for the sequelae of complex trauma at recruitment. It could be predicted that some will have unrecognised major dissociative disorders, or significant secondary or tertiary dissociative symptoms. Treatment of comorbid conditions – or concomitant symptoms – is an inadequate response to a range of complex presentations aetiologically related to early trauma.

## Therapy for severe complex PTSD and dissociative disorders

It could be argued that psychotherapy for the residual effects of trauma should start with the aim of helping those most severely affected. Chu *et al*^25^ reviewed the treatment of the major dissociative disorders which are recognised to result from early attachment trauma often compounded by later sexual and/or physical abuse. The review argued that the economic cost of dissociative disorders was considerable and highlighted the priority needed for the development of effective treatments. However, dissociative disorders were frequently unrecognised as such, perhaps because of their polysymptomatic presentations, and therefore appropriate services were not provided. When treatment was adapted to address the consequences of dissociative defences to complex trauma, even those with severe disorders could improve. The lack of controlled or randomised outcomes studies for the psychotherapy of dissociative disorders is an effect of the complexity of the presentations and of the level of funding that would be required to properly evaluate treatment. The lack of evidence is not an indicator that particular approaches do not work – only that they have not been rigorously tested. Testing procedures understandably but unhelpfully prefer simple, measurable attributes for economy of scale.

## Conclusions

Patients with many trauma-based disorders are not well served by existing therapies: they will often drop out of treatment at an early stage. PTSD is an inclusive term^5^ which has precipitated much research and clinical interest. However, this categorisation has dominated research and clinical services to the detriment of the range of disorders occurring after traumatic experience.^26^ Disorders arising from extreme stress during the brain’s development and maturation need a prolonged period for recovery. The first requirement is therefore to adopt an approach which will retain patients in therapy long enough for the therapist and patient to form a shared understanding of what is happening and to find a way of working together. This way must be found to be beneficial for the patient and sufficiently tolerable for the therapist so that the therapist does not avoid it.
